# MED12 exon 2 mutations in phyllodes tumors of the breast

**DOI:** 10.1002/cam4.462

**Published:** 2015-04-13

**Authors:** Satoi Nagasawa, Ichiro Maeda, Takayo Fukuda, Wenwen Wu, Ryosuke Hayami, Yasuyuki Kojima, Ko-ichiro Tsugawa, Tomohiko Ohta

**Affiliations:** 1 Department of Translational Oncology, St. Marianna University Graduate School of MedicineKawasaki, 216-8511, Japan; 2 Division of Breast and Endocrine Surgery, Department of Surgery, St. Marianna University Graduate School of MedicineKawasaki, 216-8511, Japan; 3 Department of Pathology, St. Marianna University Graduate School of MedicineKawasaki, 216-8511, Japan

**Keywords:** Breast, MED12, mutation, phyllodes

## Abstract

Exon 2 of MED12, a subunit of the transcriptional mediator complex, has been frequently mutated in uterine leiomyomas and breast fibroadenomas; however, it has been rarely mutated in other tumors. Although the mutations were also found in uterine leiomyosarcomas, the frequency was significantly lower than in uterine leiomyomas. Here, we examined the MED12 mutation in phyllodes tumors, another biphasic tumor with epithelial and stromal components related to breast fibroadenomas. Mutations in MED12 exon 2 were analyzed in nine fibroadenomas and eleven phyllodes tumors via Sanger sequencing. A panel of cancer- and sarcoma-related genes was also analyzed using Ion Torrent next-generation sequencing. Six mutations in fibroadenomas, including those previously reported (6/9, 67%), and five mutations in phyllodes tumors (5/11, 45%) were observed. Three mutations in the phyllodes tumors were missense mutations at Gly44, which is common in uterine leiomyomas and breast fibroadenomas. In addition, two deletion mutations (in-frame c.133_144del12 and loss of splice acceptor c.100-68_137del106) were observed in the phyllodes tumors. No other recurrent mutation was observed with next-generation sequencing. Frequent mutations in MED12 exon 2 in the phyllodes tumors suggest that it may share genetic etiology with uterine leiomyoma, a subgroup of uterine leiomyosarcomas and breast fibroadenoma.

## Introduction

Phyllodes tumors of the breast, which account for 0.3–0.9% of all breast tumors, are a fibroepithelial neoplasm seen in women aged approximately 40 years (range 9–85) 1–3. Phyllodes tumors are composed of stromal and epithelial components, a feature shared with another biphasic tumor of the breast, the fibroadenoma. Phyllodes tumors are rare, comprising only 2–3% of the fibroepithelial breast tumors in contrast to the fibroadenoma, which comprises the majority of fibroepithelial tumors; however, phyllodes tumors are clinically important because they result in considerable morbidity, with rapidly growing features and the potential for recurrence and metastasis. In spite of this importance, the etiology including genetic alteration is currently unrevealed. One candidate, the somatic mutation of mediator complex subunit 12 (MED12), has recently attracted attention.

The mediator complex is involved in transcriptional regulation by mediating the connection between transcription factors and the RNA polymerase II initiation complex 4. MED12 comprises a subunit of a CDK8 submodule of the Mediator, which also contains MED13 and Cyclin C, and regulates the kinase activity of CDK8 5,6. A study of 18 uterine leiomyomas using exome sequencing identified recurrent somatic mutations in MED12 exon 2, and further investigation of 225 uterine leiomyomas revealed that, remarkably, 159 (71%) of the tumors harbored mutations 7. Whereas MED12 mutations in other positions have been identified in prostate and adrenocortical carcinomas in much lower frequency (5%) 8,9, MED12 exon 2 mutations in other tumors have been rare events. Of 1158 tumors, including mesenchymal tumors, leukemias, breast cancers, ovarian cancers, and colorectal cancers, only five somatic alterations were observed: three in uterine leiomyosarcomas (3/41, 7%) and two in colorectal cancers (2/392, 0.5%) 10. Another study investigating 1862 tumors found that MED12 mutations occur almost exclusively in uterine leiomyomas (35/67; 52.2%), but not in other tumors, except for one colon carcinoma case (0.3%) 11. The incidence of the mutations in uterine leiomyomas and leiomyosarcomas has been further confirmed by many laboratories 10,12–21. These studies suggested that the MED12 exon 2 mutations may be unique to uterine leiomyomas and a subgroup of leiomyosarcomas; however, interestingly, a recent study using exome analysis of fibroadenomas revealed highly frequent MED12 exon 2 mutations (58/98, 59%). Of 45 somatic mutations identified in fibroadenomas, the only gene that was recurrently mutated was MED12 22, suggesting that the MED12 exon 2 mutation is a major driving mutation underlying the pathogenesis of fibroadenoma; however, the status of the mutations in phyllodes tumors, related to fibroadenomas, remains to be clarified. In the present study, we asked whether the phyllodes tumors share the same mutations with fibroadenomas, similar to leiomyomas and leiomyosarcomas.

## Materials and Methods

### Tumor samples

Archival formalin-fixed paraffin-embedded (FFPE) samples of 11 cases of phyllodes tumors and nine cases of fibroadenomas obtained from patients undergoing surgical excision at the Division of Breast and Endocrine Surgery, St. Marianna University School of Medicine, during January 2009 and December 2012, were studied. This study was approved by the Institutional Review Board of St. Marianna University School of Medicine (registration number 2330). All of the phyllodes tumors analyzed were classified as borderline according to the WHO criteria.

### DNA extraction and Sanger sequencing

The genomic DNA from the FFPE samples was extracted and purified using the QIAamp DNA FFPE Tissue Kit (QIAGEN, Hilden, German) according to the manufacturer’s instructions, and a region including MED12 exon 2 (chrX:70339100-70339472) was amplified using polymerase chain reaction (PCR) with a pair of primers (5′-TGTTCTACACGGAACCCTCCTC-3′ and 5′-CTGGGCAAATGCCAATGAGAT-3′) and PrimeSTAR Max DNA Polymerase (Takara Biotechnology, Otsu, Shiga, Japan). The PCR products were purified using agarose gel electrophoresis, labeled with BigDye Terminator (Life Technologies, Carlsbad, California, United States) with bidirectional primers, and subjected to the Applied Biosystems (Applied Biosystems, Foster, California, United States) 3130 Genetic Analyzer.

### Next-generation sequencing

Targeted next-generation sequencing was carried out using the Ion Torrent Personal Genome Machine with a panel of primers, the AmpliSeq™ Comprehensive Cancer Panel (Life Technologies), which is designed for cancer- and sarcoma-related regions, as described in the Supplementary Materials and Methods.

## Results and Discussion

MED12 exon 2 mutations were identified in six of nine fibroadenomas (67%), a frequency approximately the same as that reported previously 22. Importantly, the mutations were also found in five of eleven phyllodes tumors (45%). Although the cohort is small, the high ratio suggests that phyllodes tumors may contain a higher frequency of MED12 mutations than leiomyosarcomas, which has been reported to be approximately 10% (15 of 149 cases previously reported in total) 10,16–21. The mean age and tumor size of the cases of fibroadenoma and phyllodes tumor were 42.6- and 43.6-year-old, and 2.4 and 4.7 cm, respectively (Table S1).

**Figure 1:**
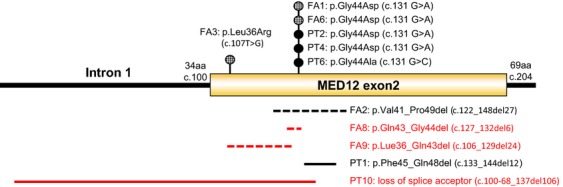
Distribution of MED12 exon 2 mutations in fibroadenomas and phyllodes tumors detected in this study. Phyllodes tumors are shown with solid circle or solid line. Fibroadenomas are shown with shaded circle and dashed line. Mutations showed in red indicate the mutations that have not been reported previously. The numbers following FA and PT are patient identification numbers in this study. del, deletion; fs, frameshift; aa, amino acid; FA, fibroadenoma; PT, phyllodes tumor.

**Figure 2:**
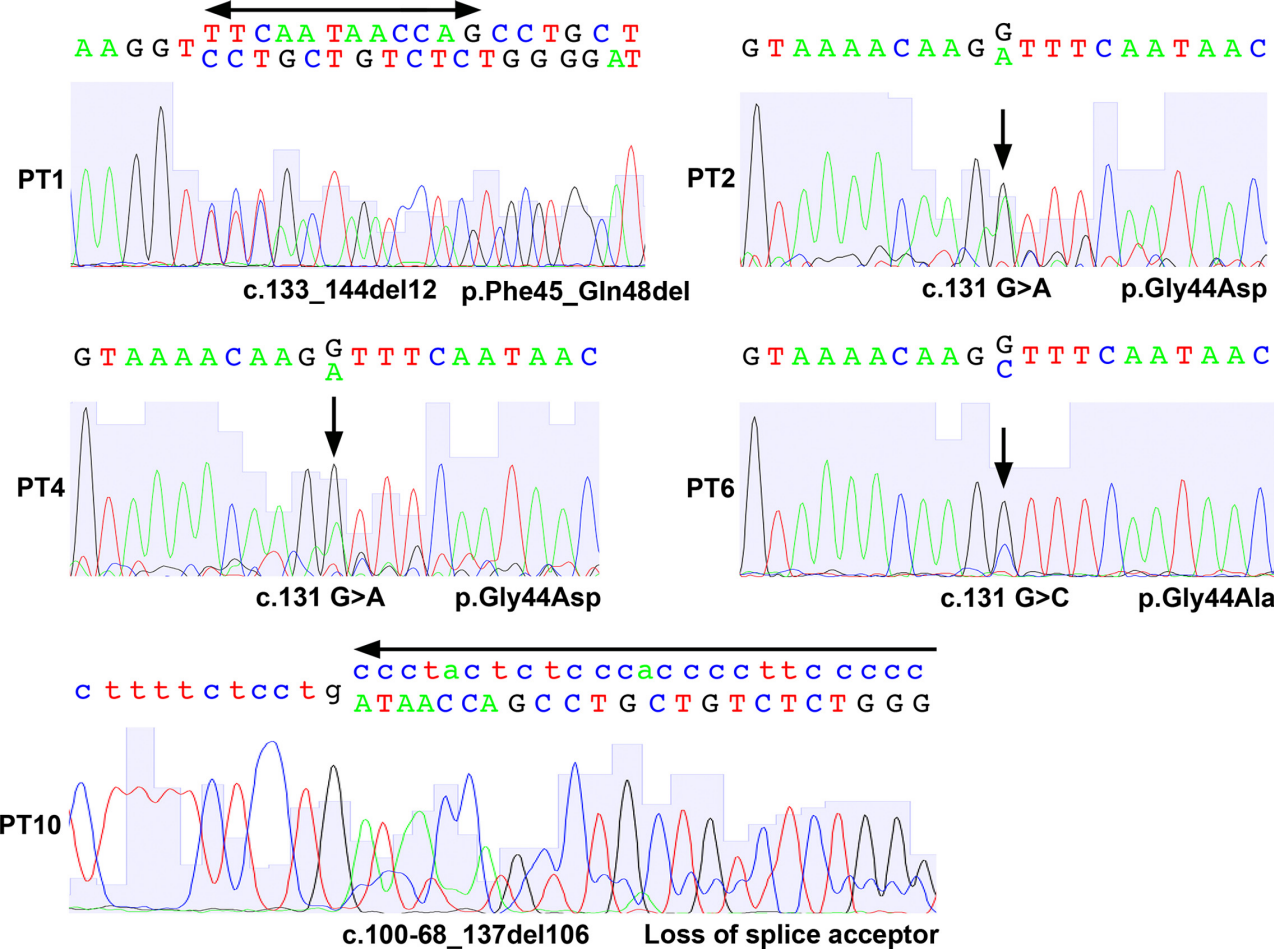
Sanger sequencing of the MED12 exon 2 mutations in phyllodes tumors. Chromatograms show the MED12 mutations in genomic DNA detected in five cases of phyllodes tumors. The arrows indicate the sites of missense mutation or deletion. Where the letters are duplicated, the upper letter(s) indicate wild type and lower letter(s) indicate variant sequences. The lower case letters indicate the nucleic acid bases in the intron. PT, phyllodes tumor case.

Two mutations in fibroadenomas (c.131 G>A; p.Gly44Asp) and three mutations in phyllodes tumors (two cases of c.131 G>A; p.Gly44Asp, and on case of c.131 G>C; p.Gly44Ala) were missense mutations in codon 44 that have been frequently mutated in uterine leiomyosarcomas and fibroadenomas (Figs.1 and 2). The codon 44 missense mutations have been observed in 42% and 49% of fibroadenomas and uterine leiomyosarcomas, respectively 7,22. The common mutation observed in fibroadenomas and phyllodes tumors suggests that these tumors may share the same genetic etiology. Other previously reported missense mutations, c.107T>G; p.Leu36Arg, and in-frame deletion, c.122_148del27; p.Val41_Pro49del, were observed in the fibroadenomas. One in-frame deletion, c.133_144del12; p.Phe45_Gln48del, previously reported for uterine leiomyoma 13,16,20, was also identified in a phyllodes tumor.

In addition, three novel deletions were identified in fibroadenomas and phyllodes tumors, including two in-frame deletions (c.127_132del6; p.Gln43_Gly44del, and c.106_129del24; p.Lue36_Gln43del) in fibroadenomas, and one relatively long deletion (c.100-68_137del106) in phyllodes tumors (Figs.1 and 2). The deletion in the phyllodes tumors started from intron 1 and included an intron–exon junction, which causes loss of the splice acceptor and therefore affects the expression of whole exon 2. All of the detected deletions affect codon 44, except one case that causes the deletion of p.Lue36_Gln43. A recent functional study revealed that the residues Leu36, Gln43, and Gly44 are each essential for the interaction of MED12 with Cyclin C-CDK8/19 complex and are required for mediator-associated CDK activity 23. Thus, the mutations identified in this study affect mediator function.

The relation of the types of mutation with the phenotypes of the tumor is currently unknown. Whereas the MED12 exon 2 mutations previously reported are mostly missense or in-frame insertion/deletion mutations, our study included a frameshift mutation and a loss of splicing acceptor mutation. Although these mutations may affect MED12 function more profoundly than the other types, they were identified both in fibroadenomas and phyllodes tumors, suggesting that the mutation types likely do not affect the malignant potential.

Although we have not performed sequencing on matched normal blood samples in this study, the mutations identified were assumed to be somatic because they are located in the common mutational hotspot observed in uterine leiomyomas and fibroadenomas, and none of the mutations were recorded as germline variants in the databases. Indeed, no germline variants in the MED12 exon 2 have been reported or are in the databases, including an analysis comprising of a predominantly east Asian or Korean cohort 11,22.

Together with the previous studies, our data suggest that the MED12 exon 2 mutation may exclusively contribute to the pathogenesis of uterine leiomyomas, leiomyosarcomas, breast fibroadenomas, and phyllodes tumors. A common feature of these tumors is an origin comprised of smooth muscle cells and estrogen- and progesterone dependency. MED12 exon 2 mutations could be implicated in hormone-dependent growth of the specific cell types, as previously proposed 22. Others have suggested that a subgroup of uterine leiomyosarcomas may develop from leiomyoma precursors 10. Similarly, our results suggest that a subgroup of phyllodes tumors is likely developed from fibroadenomas. Whereas the mutations in the leiomyosarcomas were less common than the leiomyomas, the mutations in the phyllodes tumors were more frequent in our cohort. The possible reason for the higher frequency could be due to the subtype of the phyllodes tumors investigated in this study. We selected the borderline phyllodes tumors because this subtype is likely more typical when compared to benign or malignant phyllodes tumors; however, the MED12 mutations in the malignant phyllodes tumors are a clinically important issue that remains to be investigated.

No other recurrent mutation was observed by the next-generation sequencing analyses that we performed prior to this study for the identical cases of fibroadenomas and the phyllodes tumors 24. A summary of the quality of the AmpliSeq-targeted resequencing is shown in Table S2. The exome analyses of the fibroadenomas demonstrated that the only gene that was recurrently mutated was MED12, suggesting that MED12 has a central role in fibroadenoma tumorigenesis 22. Together, our results suggest that MED12 exon 2 mutations likely have a similar impact on phyllodes tumors. In conclusion, we revealed the recurrent mutations of MED12 exon 2 in phyllodes tumors. The results suggest that a subset of phyllodes tumors may share the genetic etiology with uterine leiomyomas, fibroadenomas, and a subgroup of leiomyosarcomas.

## Supporting Information

**Figure S1.** Sanger sequencing of the MED12 exon 2 mutations in fibroadenomas. Chromatograms show MED12 mutations in genomic DNA in seven cases of fibroadenomas. The arrows indicate the sites of missense mutation or deletion. Where the letters are duplicated, the upper letter(s) indicate wild type and the lower letter(s) indicate variant sequences. The lower case letters indicate the nucleic bases in the intron. FA, fibroadenoma case.

**Table S1.** Age and tumor size of the cases analyzed for the MED12 mutations.

**Table S2.** Summary of the AmpliSeq-targeted resequencing of the phyllodes tumors and fibroadenomas.
