# Supplementary figures and images for: MED12 exon 2 mutations in phyllodes tumors of the breast
Source: Cancer Med. 2015 Apr 13;4(7):1117–21. doi: 10.1002/cam4.462 (PMC4529349; doi:10.1002/cam4.462)

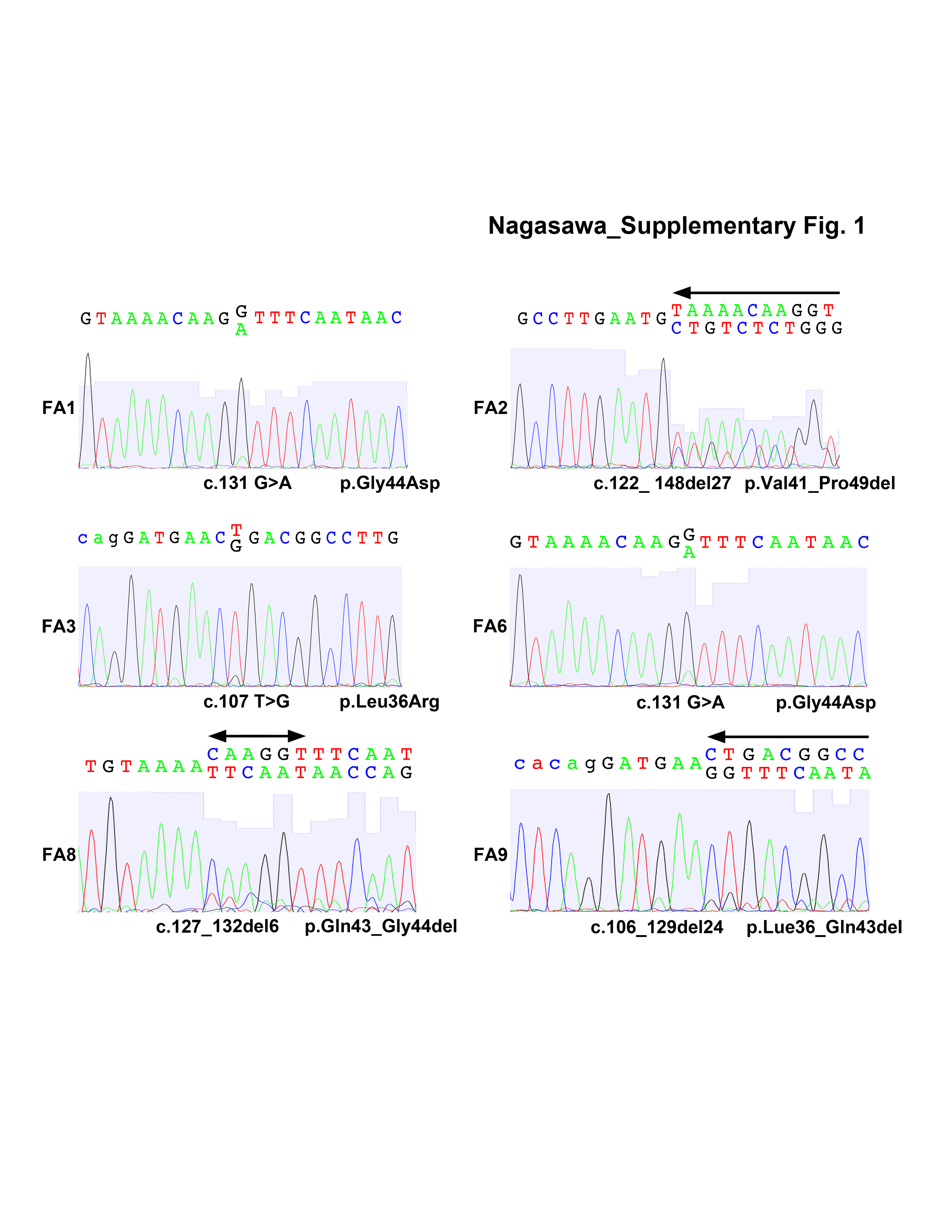

Supplement: Supplementary file 1 [file cam40004-1117-sd1.tif]
