# Supplementary material for: MED12 exon 2 mutations in phyllodes tumors of the breast
Source: Cancer Med. 2015 Apr 13;4(7):1117–21. doi: 10.1002/cam4.462 (PMC4529349; doi:10.1002/cam4.462)
Supplement: Supplementary file 2 [file cam40004-1117-sd2.docx]

**Supplementary figure legend**

**Supplementary Figure 1.** Sanger sequencing of the MED12 exon 2 mutations in fibroadenomas. Chromatograms show MED12 mutations in genomic DNA in seven cases of fibroadenomas. The arrows indicate the sites of missense mutation or deletion. Where the letters are duplicated, the upper letter(s) indicate wild type and the lower letter(s) indicate variant sequences. The lower-case letters indicate the nucleic bases in the intron. FA, fibroadenoma case.

**Supplementary materials and methods**

**Next-generation sequencing**

Multiplex PCR was achieved using the Ion AmpliSeq™ Library Kit 2.0 with the DNA sample (10 ng) and primers of a AmpliSeq™ Comprehensive Cancer Panel (Life Technologies), which is designed for cancer- and sarcoma-related 15,992 regions in 409 genes not involving MED12, according to the manufacturer’s instructions. The amplicons from each case were labeled with a barcode adapter, mixed, and further amplified by emulsion PCR using the Ion PGMTM Template OT2 200 Kit (Life Technologies). The library DNA was annealed with sequencing primers and mixed with DNA polymerase and dNTPs from the Ion PGM^TM^ Sequencing 200 Kit v2 (Life Technologies), and loaded onto the Ion Torrent Personal Genome Machine (PGM; Life Technologies) with 316 chips. Sequence alignment and variant calling was performed using CLC Genomics Work Bench 6 (CLC bio) with the hg19 human reference genome. The variants were flagged when the position was covered at least 10 times. The candidate variants were further analyzed by Torrent Suite v4.0.1 (Life Technologies), dbSNPs (data base single nucleotide polymorphisms), 1000 Genomes Project, and COSMIC (COSMIC: Catalogue Of Somatic Mutations In Cancer). The alignments were visually verified using the CLC Genomics Work Bench 6.
