# Supplementary material for: MED12 exon 2 mutations in phyllodes tumors of the breast
Source: Cancer Med. 2015 Apr 13;4(7):1117–21. doi: 10.1002/cam4.462 (PMC4529349; doi:10.1002/cam4.462)
Supplement: Supplementary file 3 [file cam40004-1117-sd3.docx]

Supplementary Table1. Age and tumor size of the cases analyzed for the MED12 mutations.

Case Age tumor size (cm) Mutation identified

FA1 22 2.5 c.131 G>A; p.Gly44Asp

FA2 44 3.0 c.122_148del27; p.Val41_Pro49del

FA3 45 2.7 c.107T>G; p.Leu36Arg

FA4 65 1.6

FA5 39 1.0

FA6 37 2.6 c.131 G>A; p.Gly44Asp

FA7 47 3.0

FA8 46 2.4 c.127_132del6; p.Gln43_Gly44del

FA9 38 1.9 c.106_129del24; p.Lue36_Gln43del

average 42.6 2.4

PT1 58 9.5 c.133_144del12; p.Phe45_Gln48del

PT2 60 4.0 c.131 G>A; p.Gly44Asp

PT2 23 4.5

PT4 35 3.7 c.131 G>A; p.Gly44Asp

PT5 44 2.4

PT6 36 12.0 c.131 G>C; p.Gly44Ala

PT7 29 3.0

PT8 46 3.0

PT9 48 3.8

PT10 46 2.5 c.100-68_137del106; Loss of splice acceptor

PT11 57 3.5

average 43.6 4.7
