# Supplementary material for: MED12 exon 2 mutations in phyllodes tumors of the breast
Source: Cancer Med. 2015 Apr 13;4(7):1117–21. doi: 10.1002/cam4.462 (PMC4529349; doi:10.1002/cam4.462)
Supplement: Supplementary file 4 [file cam40004-1117-sd4.docx]

Suppementaly Table2. Summary of the AmpliSeq targeted resequencing of the phyllodes tumors and fibroadenomas.

| Specimen ID | Bases in Target Region | Reads Mapped to Target Region | Ave. Depth Per Targeted Base | Targeted Bases with Depth at Least 1X(%) | Targeted Bases with Depth at Least 20X(%) |
| --- | --- | --- | --- | --- | --- |
| FA1 | 1,688,650 | 256,613 | 15.8 | 96.9 | 30.5 |
| FA2 | 1,688,650 | 213,127 | 12.8 | 95.7 | 19.6 |
| FA3 | 1,688,650 | 231,192 | 14.1 | 96.7 | 23.6 |
| FA4 | 1,688,650 | 208,438 | 12.5 | 93.4 | 20.8 |
| FA5 | 1,688,650 | 266,130 | 15.7 | 89.3 | 31.4 |
| FA6 | 1,688,650 | 338,103 | 20.3 | 97.3 | 43.2 |
| FA7 | 1,688,650 | 224,572 | 13.4 | 96.8 | 21.1 |
| FA8 | 1,688,650 | 348,080 | 20.9 | 97.3 | 46.4 |
| FA9 | 1,688,650 | 287,860 | 17.2 | 96.9 | 34.8 |
| Average | 1,688,650 | 263,791 | 15.9 | 95.6 | 30.2 |
| PT1 | 1,688,650 | 269,385 | 14.3 | 93.7 | 22.8 |
| PT2 | 1,688,650 | 385,566 | 21.1 | 96.6 | 41.0 |
| PT3 | 1,688,650 | 365,841 | 20.6 | 79.3 | 40.5 |
| PT4 | 1,688,650 | 26,498 | 1.4 | 53.3 | 0.1 |
| PT5 | 1,688,650 | 338,060 | 18.6 | 76.5 | 36.1 |
| PT6 | 1,688,650 | 647,254 | 36.1 | 94.8 | 52.7 |
| PT7 | 1,688,650 | 1,036,442 | 59.2 | 80.1 | 65.7 |
| PT8 | 1,688,650 | 2,714 | 0.1 | 8.7 | 0.1 |
| PT9 | 1,688,650 | 386,177 | 18.7 | 94.9 | 29.6 |
| PT10 | 1,688,650 | 308,112 | 17.3 | 95.3 | 29.3 |
| PT11 | 1,688,650 | 150,745 | 9.0 | 95.4 | 7.3 |
| Average | 1,688,650 | 356,072 | 19.7 | 79.0 | 29.6 |
